# Long-term cognitive outcomes in term newborns with watershed injury caused by neonatal encephalopathy

**DOI:** 10.1038/s41390-021-01526-2

**Published:** 2021-10-26

**Authors:** Bo Lyun Lee, Dawn Gano, Elizabeth E. Rogers, Duan Xu, Stephany Cox, A. James Barkovich, Yi Li, Donna M. Ferriero, Hannah C. Glass

**Affiliations:** 1 Department of Pediatrics, Busan Paik Hospital, Inje University College of Medicine, Busan, Korea; 2 Department of Neurology, University of California San Francisco, San Francisco, CA USA; 3 Department of Pediatrics, University of California San Francisco, San Francisco, CA USA; 4 Department of Radiology and Biomedical Imaging, University of California San Francisco, San Francisco, CA USA; 5 Department of Epidemiology and Biostatistics, University of California San Francisco, San Francisco, CA USA

## Abstract

**Background:**

We previously reported that increasing severity of watershed (WS) injury in neonatal magnetic resonance imaging (MRI) is associated with worse language outcomes in early childhood. In the present study, we investigated the relationship between neonatal injury patterns and cognitive profile in adolescents with neonatal encephalopathy.

**Methods:**

Term neonates with encephalopathy were prospectively enrolled and imaged using brain MRI from 1999 to 2008. Neonatal brain injury was scored according to the degree of injury in WS and basal ganglia/thalamus (BG/T) areas. The children underwent a neurocognitive assessment and follow-up brain MRI at the age of 10–16 years. The relationship between neonatal brain injury patterns and adolescent cognitive outcomes was assessed.

**Results:**

In a cohort of 16 children, neonatal MRI showed WS injury in 7, BG/T injury in 2, and normal imaging in 7. Children with WS injury had lower estimated overall cognitive ability than those with normal imaging. Increasing WS injury score was associated with decreasing estimated overall cognitive ability, Perceptual Reasoning Index, and digit span score.

**Conclusions:**

Children with the WS injury are at an increased risk of having problems in long-term intellectual ability. These cognitive outcomes may underlie early language difficulties seen in children with neonatal WS injury.

**Impact:**

- Adolescents with a history of neonatal encephalopathy and watershed pattern of injury on neonatal brain magnetic resonance imaging (MRI) had lower overall cognitive ability, perceptual reasoning skills, and auditory working memory than those with normal neonatal imaging.
- Children with post-neonatal epilepsy and cerebral palsy had the worst cognitive outcomes.
- Watershed pattern of injury confers high long-term differences in intellectual ability.

## Introduction

Neonatal encephalopathy due to hypoxic–ischemic insults is the most common cause of brain injury in term newborns. It has the incidence of approximately 1.5 cases per 1000 live births in high-income countries and 10–20 cases per 1000 live births in low- and middle-income countries.^1–3^ A high prevalence of long-term neurologic morbidities such as cerebral palsy, epilepsy, intellectual disability, and behavioral disorders has been reported.^4,5^ Cognitive and behavioral problems after neonatal encephalopathy have been reported in children without cerebral palsy and severe intellectual disabilities.^6–8^ Neonatal brain magnetic resonance imaging (MRI) is the best available tool to identify specific brain regions involved, and the degree of brain injury in infants with neonatal encephalopathy, as well as to predict outcomes in infancy and childhood.^9–12^ We previously reported that a predominant basal ganglia/thalamus (BG/T) injury pattern showed worse cognitive and functional motor outcomes than did a watershed (WS) injury pattern at the age of 30 months, and the WS injury pattern can be associated with cognitive impairment without neuromotor deficits.^13^ At the age of 4 years, an increasing WS injury pattern was associated with a greater deficit in verbal intelligence quotient (IQ) among newborns without motor deficits.^8^ We recently reported that local changes in the volume of perisylvian gray and white matter at the age of 6 months are associated with adverse language outcomes at the age of 30 months in survivors of neonatal encephalopathy.^14^

Several studies have reported early school-age cognitive and neurological outcomes in term newborns with moderate-to-severe hypoxic–ischemic injury.^5,15,16^ However, little is known about the outcomes in adolescence. A few studies have reported that cognitive problems can occur in children at late school age depending on the clinical severity of neonatal encephalopathy but the relation with neonatal neuroimaging was not well evaluated.^17–19^ One prior study has reported that the severity of WS injury was associated with later intellectual performance in adolescence.^7^

In the present study, we examined the association between the severity and pattern of neonatal brain injury and cognitive outcomes in adolescence in those with a history of neonatal encephalopathy caused by presumed hypoxic–ischemic insult. We hypothesized that the adolescents with a history of WS injury would have worse cognitive outcomes than those with normal neonatal imaging.

## Methods

### Subjects

This was a sub-study of the previously described prospective cohort study (the Birth Asphyxia MRI or BAMRI study) of newborns beyond 36 weeks of gestation who presented with neonatal encephalopathy caused by presumed hypoxic–ischemic insult and were admitted to the Intensive Care Nursery at the University of California, San Francisco (UCSF) from study initiation in 1999 through 2008.^8^ The protocol for the ongoing study was approved by the Committee on Human Research at the UCSF, and the subjects were enrolled in the study only after voluntary informed consent was obtained from the parents and assent from the adolescent participants.

All enrolled participants had one or more of the following markers of neonatal encephalopathy: (a) 5-min Apgar score of ≤5; (b) umbilical artery cord blood pH <7.1; (c) umbilical artery base deficit ≤−10; and/or (d) clinical brain dysfunction (defined by abnormal tone, feeding, alertness, respiratory status, and/or reflexes). Newborns with gestational age <36 weeks and those with suspected or confirmed congenital malformations, inborn errors of metabolism, or congenital infections based on clinical examination and laboratory studies were excluded from the study. The severity of neonatal encephalopathy was graded from zero (no encephalopathy) to seven (severe encephalopathy) using a previously published scoring system based on the state of consciousness, tone, respiratory status, feeding, crying, reflexes, and seizure activity.^20^ The resuscitation score at birth was measured and graded from one (no intervention) to six (endotracheal intubation with ventilation and sodium bicarbonate with or without epinephrine) using a previously described scoring system.^13^ Neonatal seizures were graded according to the severity (0–11) using a scoring system based on the day of onset, number of episodes, birth electroencephalography, and number of anti-seizure drugs administered during hospital stay.^21^ No participant was treated with therapeutic hypothermia. Inclusion criteria for the participants in the present study were as follows: (1) alive and aged 10–16 years during the study period; (2) able to undergo MRI without sedation; (3) underwent neuropsychological testing using either Wechsler Intelligence Scale for Children Fourth or Fifth Edition (WISC-IV, V) or Wechsler Abbreviated Scale of Intelligence Second Edition (WASI-II); and (4) participant parents were literate in English or Spanish language. The child and parents were invited to the UCSF campus to undergo MRI, neuropsychological testing, and neurological examination during a single visit.

### Measurements

#### Brain MRI

MRIs were acquired using a 1.5-Tesla GE MRI scanner during the first 2 weeks of life and 3.0-Tesla GE MR750 during adolescence. The neonatal brain MRI protocol comprised a standard set of sequences including T1-weighted (two-dimensional (2D) spin-echo echo time [TE]/repetition time [TR]: 11/500 ms, slice thickness: 4 mm, skip: 1 mm), T2-weighted (2D dual spin echo TE: 60/120 ms, TR: 3 s, slice thickness: 4 mm, skip: 2 mm), and diffusion-weighted images. During adolescence, the MRI protocol comprised T1-weighted images that were acquired using sagittal three-dimensional inversion recovery spoiled gradient echo (TR: minimum, TE: minimum, inversion time = 450.00 ms) yielding images with isotropic 1 × 1 × 1 mm^3^ spatial resolution and T2-weighted (2D spin echo TE/TR 120/3000 ms, slice thickness: 2 mm, no skip) images.

A neuroradiologist blinded to the participants’ clinical condition reviewed the MRI scans. The severity of neonatal brain injury in the WS distribution and BG/T distribution was scored from zero to five and zero to four, respectively, based on a previously published scoring system for acute and subacute signal abnormalities.^8,9^ Adolescent MRIs were also reviewed by the neuroradiologist. The inter-observer and intra-observer reliability of MRI scores in this cohort have been previously reported with a kappa of 0.85 and 0.85–1.0, respectively.^9,13^

#### Neurodevelopmental evaluation

The participants were followed-up longitudinally at 24 months, 4 years, 8 years, and between 10 and 16 years of age. They underwent neurocognitive assessments using Bayley Scales of Infant Development, Second Edition (Bayley-II) or Bayley Scales of Infant and Toddler Development, Third Edition (Bayley-III) at 24 months of age; Wechsler Preschool and Primary Scale of Intelligence-Revised (WPPSI-R) at 4 years of age; WISC-IV at 8 years of age, and WISC-IV, V, or WASI-II during adolescence. The assessments were performed by a clinical psychologist blinded to participant neonatal course and MRI findings.

Estimated full-scale IQ, Verbal Comprehension Index, and Perceptual Reasoning Index were evaluated using WASI-II and WISC-IV. Scaled scores of digit span and coding were assessed for auditory working memory index and processing speeding index, respectively, using WISC-IV and V. General ability index was calculated using WISC-IV and was used as an estimated full-scale IQ. General ability index on WISC-IV and estimated IQ on WASI-II were referred to as estimated overall cognitive ability. The results of WPPSI-R, WISC-IV, and WASI-II are expressed as an age-standardized score, with a mean of 100 and a standard deviation of 15. The cognitive scores of Bayley-II were expressed as Mental Developmental Index and Bayley-III as Cognitive Composite and Language Composite (CL-III) at 24 months; the CL-III is defined as the average score of Cognitive Composite scale and Language Composite scale of Bayley-III.^22^

A standardized, detailed neurologic examination was performed by a pediatric neurologist who was blinded to the neonatal course and imaging results. The neurologist scored neuromotor outcomes with a previously published score as follows: zero = normal, one = abnormal tone or reflexes, two = abnormal tone and reflexes, three = decreased power in addition to tone or reflex abnormality (functional deficit of power), four = cranial nerve involvement with motor abnormality, and five = spastic quadriparesis.^23^ Presence and pattern of cerebral palsy and Gross Motor Function Classification System (GMFCS)^24^ were determined based on review of the detailed neurological examination.

### Statistical analyses

All statistical analyses were performed using SPSS for Windows, version 25.0 (SPSS Inc., an IBM Company, Chicago, IL, USA). Continuous variables are presented as mean ± standard deviation or median (interquartile range) for variables that were not normally distributed, and qualitative variables are expressed as percentages. Fisher’s exact test was used for comparing categorical variables. Student’s *t* test, Kruskal–Wallis test, and analysis of variance were used to compare continuous variables. Two-tailed null hypotheses of no difference were rejected if *p* values were <0.05. The association between the severity of WS injury and cognitive profile in adolescence was evaluated using Spearman’s rank correlation test.

## Results

### Neonatal clinical characteristics

**Fig. 1:**
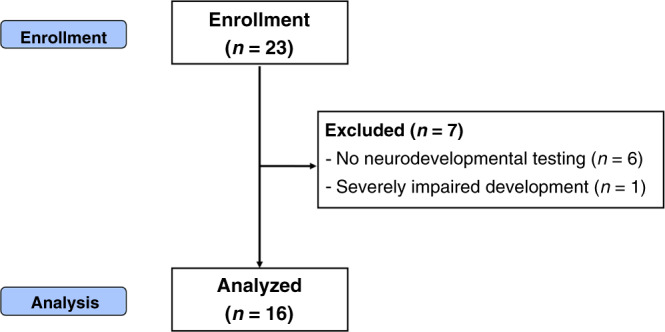
Flow diagram describing the study population.

Twenty-three children were scheduled for follow-up procedures, of whom 16 successfully completed both MRI and neurocognitive testing in adolescence (Fig. 1). The WS predominant pattern of injury was present in 7/16 (44%), BG/T predominant injury in 2/16 (13%), and normal scan in 7/16 (44%) neonatal MRIs. Two children with BG/T predominant pattern of injury also had anterior or posterior white matter injury. Seven children with WS injury did not show deep gray nuclei injury.

Newborns with WS or BG/T injury had lower birth weight and higher seizure score than the newborns with normal brain imaging (Table 1). No other significant differences in baseline demographic characteristics were observed among the three groups.

**Table 1:** Clinical characteristics of the participants with neonatal encephalopathy classified by brain MRI pattern.

Variables	Normal (*n* = 7)	WS injury (*n* = 7)	BG/T injury (*n* = 2)	*p*
*Baseline characteristics*				
Gestational age—weeks	40.3 ± 1.5	39.0 ± 2.0	38.2 ± 2.6	0.33
Birth weight—g	3771 ± 474	3095 ± 473	3080 ± 891	0.07
Male sex—no. (%)	3 (43)	6 (86)	1 (50)	0.30
Emergency cesarean delivery—no. (%)	2 (29)	4 (57)	0 (0)	0.48
Apgar score at 5 min—median (IQR)	7 (5–8)	5 (4–7)	5 (3, 7)^**a**^	0.36
Encephalopathy score—median (IQR)	2 (1–6)	5 (3–5)	6 (6, 6)	0.14
Resuscitation score—median (IQR)	4 (4–5)	4 (4–5)	5 (4, 6)	0.38
Seizure score—median (IQR)	0 (0–0)	2 (0–3)	4 (3, 4)	0.003
UCAB—pH	7.02 ± 0.19	7.00 ± 0.176	7.00 ± 0.11	0.96
UCAB Base deficit—mmol/L	−11.2 ± 4.0	−14.1 ± 5.8	−12.5 ± 0.71	0.57
Maternal education some college or higher—no. (%)	7 (100)	5 (83)	2 (100)	0.53
*Follow-up in adolescence*				
Age at exam—years	13.6 ± 1.7	11.9 ± 1.9	13.1 ± 3.6	0.31
Height—cm	163 ± 10	144 ± 25	155 ± 31	0.24
Body weight—Kg	55 ± 12	44 ± 15	48 ± 22	0.40
Head circumference—cm	56 ± 1	55 ± 3	55 ± 3	0.87
Neuromotor score—median (IQR)	0 (0–1)	0 (0–1)	3 (0, 5)	0.14
Score on Gross Motor Function Classification System—median (IQR)	0 (0–0)	0 (0–0)	2 (0, 4)	0.03

Data are presented as median (IQR), mean ± standard deviation or number (%). *p* values refer to comparisons across the three groups.

*BG/T* basal ganglia/thalamus, *IQR* interquartile range, *UCAB* umbilical cord arterial blood, *WS* watershed.

^a^The data of children with BG/T are presented as median (minimum value, maximum value).

### Follow-up in adolescence

#### Clinical evaluation

Follow-up evaluation was done at the median age of 13 (range, 10.3–15.6) years. No significant differences were observed in height, weight, and head circumference among the brain injury groups and normal imaging group (Table 1). Children with BG/T predominant pattern of injury had a higher (worse) median neuromotor score than other groups, although the difference was not significant in this small cohort. At the time of follow-up, two children had clinical diagnoses of cerebral palsy, epilepsy, and low cognition (Table 2). Two children were diagnosed with cerebral palsy: ataxic/dystonic cerebral palsy (GMFCS IV) in a child with injury to the posterior putamina and ventrolateral thalami bilaterally (patient no. 13), and spastic triplegia (GMFCS I) in a child with supratentorial white matter injury (patient no. 14). One child with WS pattern of injury had clinical diagnoses of attention deficit hyperactivity disorder, learning disability, and sensorineural hearing loss.

**Table 2:** Neonatal and follow-up characteristics of 16 children with neonatal encephalopathy and adolescent outcome.

Patient no.	Neonatal MRI			Neurodevelopmental evaluation in childhood			MRI in adolescence		Neurodevelopmental evaluation in adolescence		
	Predominant pattern of injury	WS score	BG/T score	Bayley-II MDI or Bayley-III CL-III^a^ at 2 years	WPPSI-R Full-scale IQ at 4 years	WISC-IV Full-scale IQ at 8 years	Age at follow-up (years)	Pattern	Clinical neurological diagnoses	NMS	Overall cognitive ability^b^
1	WS	2	0	93	107	ND	15.0	Normal	None	0	100
2	Normal	0	0	95	99	109	14.6	Normal	None	0	100
3	Normal	0	0	99	111	99	15.0	Normal	None	0	111
4	Normal	0	0	105	80	98	14.9	Normal	None	0	103
5	Normal	0	0	114	107	103	14.3	Normal	None	0	116
6	Normal	0	0	110	109	109	13.8	Normal	None	1	120
7	WS	1	0	50	89	80	14.1	Decreased WM in the right temporal area	None	1	87
8	BG/T	2	3	102	110	129	15.6	Focal signal abnormality in left thalamus and bilateral occipital lobes	None	0	119
9	WS	1	0	89	110	104	12.1	Subtle signal abnormality in bilateral posterior putamina	None	1	113
10	Normal	0	0	108	ND	119	12.0	Subtle narrowing of the posterior limb of internal capsule	Glasses for myopia	1	123
11	Normal	0	0	87	119	ND	10.6	Normal	None	0	120
12	WS	4	0	85	80	89	10.3	Diffuse decreased WM volume	Learning difficulty, ADHD, SNHL, glasses for myopia	0	89
13	BG/T	2	2	71	81	ND	10.6	Thalamus and putamina injury, left temporal WM lesion	Cerebral palsy, epilepsy, borderline cognition	5	73
14	WS	3	0	77	51	ND	10.4	Extensive WM injury, thinning of cerebral cortex and corpus callosum	Cerebral palsy, epilepsy, intellectual disability	3	51
15	WS	4	0	101	112	ND	11.2	Focal signal abnormality in left occipital lobe	None	0	114
16	WS	2	0	97	109	ND	10.3	Subtle WM injury near left lateral ventricle	None	0	101

*ADHD* attention deficient hyperactivity disorder, *BG/T* basal ganglia/thalamus, *Bayley-II* Bayley Scales of Infant Development, second edition, *Bayley-III* Bayley Scales of Infant and Toddler Development, Third Edition, *CL-III* Cognitive and Language Composite of Bayley-III, *MDI* Mental Developmental Index, *ND* not done, *NMS* neuromotor score, *SNHL* sensory neural hearing loss, *WISC-IV* Wechsler Intelligence Scale for Children Fourth, *WPPSI-R* Wechsler Preschool and Primary scale of Intelligence-revised, *WM* white matter, *WS* watershed.

^a^The Cognitive and Language Composite (CL-III) is defined as the average score of the Cognitive Composite and the Language Composite scales of Bayley-III. The Bayley-II is applied to patient no. 1–11, and the Bayley-III in patient no. 12–16.

^b^Estimate of overall cognitive ability represents general ability index converted from WISC-IV full-scale IQ (*n* = 10) and estimated full-scale IQ of Wechsler Abbreviated Scale of Intelligence (WASI), Second Edition (*n* = 6).

#### Adolescent MRI

**Fig. 2:**
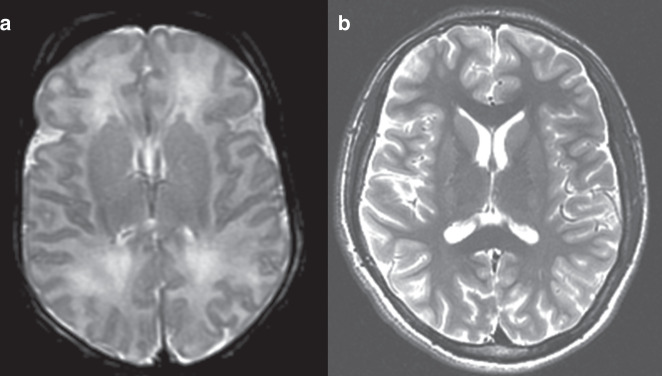
Neonatal and adolescent magnetic resonance imaging (MRI) of patient no. 1. **a** Axial T2-weighted imaging of the neonatal brain demonstrates T2-hyperintensity in bilateral frontal and parietal white matter in a watershed distribution. There was no associated reduced diffusion (not shown), so injury appears subacute. **b** Follow-up adolescent MRI through the same level demonstrates normal signal and volume.

**Fig. 3:**
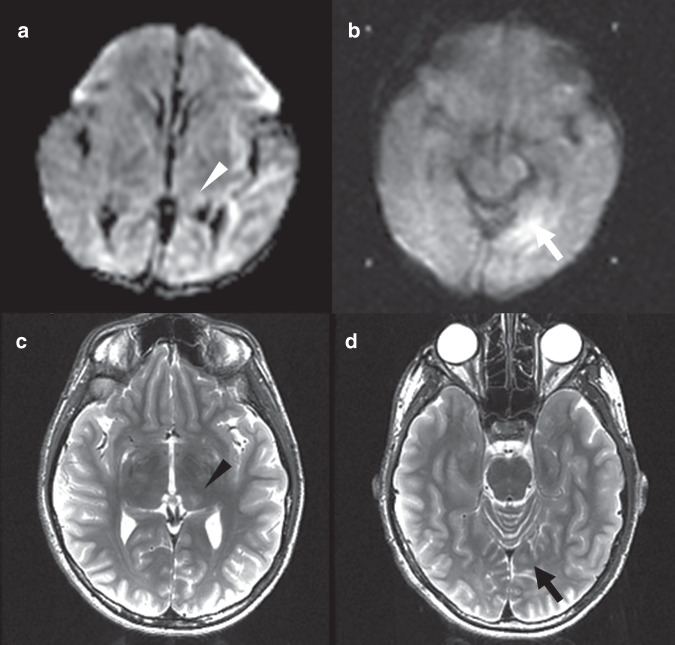
Neonatal and adolescent magnetic resonance imaging (MRI) of patient no 8. **a** Axial diffusion-weighted imaging of the neonatal brain demonstrates reduced diffusion in the left thalamus (white arrowhead). **b** Additional area of reduced diffusion in a watershed distribution in the medial left occipital lobe on neonatal MRI (white arrow). **c** Axial T2-weighted imaging in adolescent MRI demonstrates T2 hyperintensity and volume loss in the left thalamus (black arrowhead), corresponding to area of reduced diffusion in **a**. **d** T2 hyperintensity in the medial left occipital subcortical white matter (black arrow) corresponding to area of reduced diffusion in **b**.

In 8/9 (89%) children with brain injury on neonatal imaging, adolescent MRI showed expected evolution of neonatal injury in terms of location and pattern of injury, and there were no other underlying or newly occurring problems. One child (patient no. 1) with neonatal WS injury had normal brain imaging in adolescence (Fig. 2), and his development and cognition were normal (Table 2). In another child (patient no. 8), adolescent MRI showed evolution of thalamic and WS injury seen on neonatal MRI (Fig. 3). The patient was classified as having BG/T predominant pattern of injury because the BG/T scores were higher than the WS scores, but he also had WS injury in bilateral occipital lobe. Reduced diffusion in bilateral posterior insular cortex in neonatal imaging improved in adolescent follow-up MRI. Seven children with normal neonatal imaging also presented with normal imaging in their adolescent MRI except for one child who showed subtle narrowing of the posterior limb of the bilateral internal capsules.

### Neurodevelopmental outcome

**Fig. 4:**
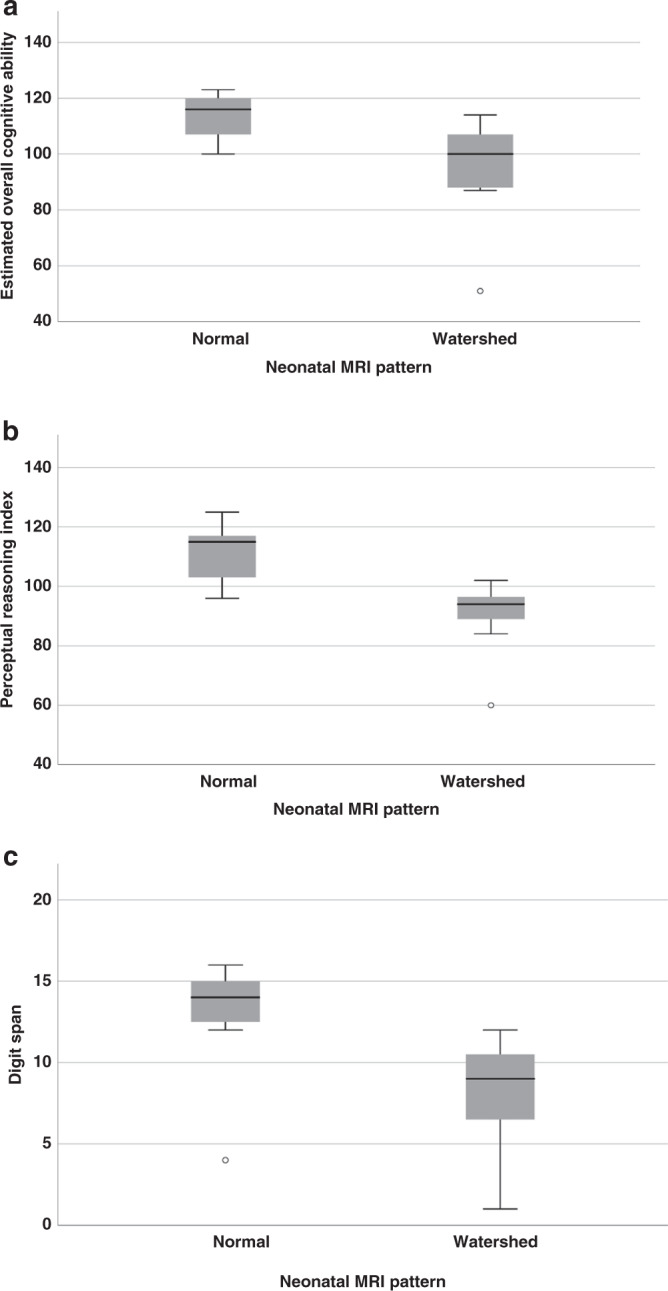
Cognitive outcomes in participants stratified by normal finding and watershed injury pattern in neonatal brain MRI. **a** Standard score of estimated overall cognitive ability (*p* = 0.04). **b** Standard score of Perceptional Reasoning Index (*p* = 0.007). **c** Scaled score of digit span (*p* = 0.04).

Among 16 adolescent participants with neuropsychological evaluations, WASI-II and WISC-V were performed in six participants and WISC-IV in 10. Overall, 9/16 (56%) participants had overall cognitive ability in the average range (85–115), 2/16 (13%) below average, and 5/16 (31%) above average. Overall cognitive ability was normal in all children with normal neonatal imaging. The mean estimate of overall cognitive ability in children with WS pattern of injury was lower than those with normal imaging (94 ± 21 vs. 113 ± 9, *p* = 0.04; Table 3 and Fig. 4). In 2 children with BG/T pattern of injury, the estimate of overall cognitive ability was 119 and 73. Additionally, mean Perceptual Reasoning Index was lower in children with WS pattern of injury than those with normal imaging (90 ± 14 vs. 111 ± 10, *p* = 0.007). Children with WS pattern of injury also had lower Verbal Comprehension Index than those with normal imaging although the difference was not significant (97 ± 27 vs. 111 ± 9, *p* = 0.21). When excluding the outlying participant with an IQ of 51, the association between WS pattern of injury and lower overall cognitive ability and Perceptual Reasoning Index was unchanged.

**Table 3:** Cognitive outcomes of participants in adolescence.

	All participants (*n* = 16)	Normal (*n* = 7)	WS injury (*n* = 7)	*p*
*Estimate of overall cognitive ability*				
Standard score	103 ± 20	113 ± 9	94 ± 21	0.04
*Verbal Comprehension Index*				
Standard score	103 ± 20	111 ± 9	97 ± 27	0.21
*Perceptual Reasoning Index*				
Standard score	102 ± 17	111 ± 10	90 ± 14	0.007
*Digit span*				
Scaled score	11 ± 4	13 ± 4	8 ± 4	0.04
*Coding*				
Scaled score	10 ± 5	12 ± 4	8 ± 5	0.16

Data are presented as mean ± standard deviation.

*WS* watershed.

The 4-year neurodevelopmental assessment was associated with adolescent cognitive outcome; higher Full-scale IQ and Verbal IQ at age 4 years were associated with higher estimated overall cognitive ability (Spearman’s *r* = 0.763, *p* = 0.002) and Verbal Comprehension Index (Spearman’s *r* = 0.849, *p* = 0.000) in adolescence, respectively. Performance IQ showed weak relationship with Perceptual Reasoning index (Spearman’s *r* = 0.505, *p* = 0.07).

Increasing severity of WS injury was significantly correlated with a lower estimated overall cognitive ability index (Spearman’s *r* = −0.558, *p* = 0.04), lower Perceptual Reasoning Index (Spearman’s *r* = −0.722, *p* = 0.004), and lower digit span scores (Spearman’s *r* = −0.698, *p* = 0.006).

## Discussion

In the present cohort study of adolescents with a history of neonatal encephalopathy, we showed that children with a WS pattern of injury on neonatal imaging had a lower overall cognitive ability at the age of 10–16 years as compared to those who had a normal neonatal MRI. Increasing severity of WS injury was significantly associated with decreasing overall cognitive abilities. The participants with comorbid diagnosis of post-neonatal epilepsy and cerebral palsy showed the lowest cognition in this cohort.

These findings are consistent with those of the prior studies examining the relationship between early imaging and childhood and adolescent outcomes following neonatal encephalopathy. We previously showed that the severity of WS injury was associated with a lower verbal IQ at the age of 4 years in children without motor deficits.^8^ Barnett et al.^25^ reported that 80% of the children, aged 5–6 years with minor neurological dysfunction or perceptual–motor difficulties had a mild-to-moderate BG/T injury or marked WS lesion on their neonatal MRI. Van Kooij et al.^26^ reported that moderate-to-severe injury (WS injury, BG/T injury, or focal infarction) observed on neonatal and childhood MRI was associated with an impaired cognitive outcome at the age of 9–10 years in children with a history of neonatal encephalopathy.

We also showed results of adolescent follow-up MRI in addition to neonatal imaging. Adolescent MRI had the expected evolution of neonatal brain injury in most children (eight out of nine children, 89%), and there was no additional etiology found. However, one child with neonatal WS white matter injury showed normal brain imaging without evolving into more serious injury in adolescence, and normal development and cognition was observed. Van Kooij et al.^26^ also reported that three out of seven children with white matter injury due to neonatal encephalopathy in neonatal MRI had normal childhood imaging. The implications of normalization of apparent white matter injury in the setting of neonatal encephalopathy are uncertain and merit additional investigation.

Our findings also support prior studies that suggest persistent difficulties with cognitive function in adolescence. Lindström et al.^19^ evaluated 28 adolescents at the age of 15–19 years for cognitive and behavioral outcomes who were born in Sweden with a history of moderate neonatal encephalopathy without cerebral palsy or major neurologic impairments (ataxia and severe mental retardation); their siblings (*n* = 15) without hypoxic–ischemic insult comprised the control group. Twenty teenagers among 28 (71%) had cognitive dysfunctions, such as low or borderline IQ and learning disabilities, which were more prevalent than their siblings (20/28 [71%] vs. 2/15 [13%]). Problems of short-term memory, time perception, and orientation were more frequently reported in these adolescents with a history of neonatal encephalopathy as compared to their siblings. Perez et al.^7^ evaluated the neurocognitive outcomes in 57 children with a history of neonatal hypoxic–ischemic encephalopathy who were devoid of major disabilities (cerebral palsy or intellectual disability) at a median age of 11 years (range, 8–16 years). In this cohort, full-scale IQ and performance IQ scores were significantly lower than the population norm. They reported an association between higher WS injury score and lower full-scale IQ and verbal IQ. Our results are in line with Perez’s report in terms of the association between overall cognitive ability and WS pattern of injury on neonatal MRI. In our participants with WS injury, only one child had functional motor deficits. Verbal Comprehension Index scores were also lower in children with WS injury than those with normal imaging although the mean scores were not significantly different in a small cohort. Additionally, we observed a trend toward increasing severity of WS pattern of injury and decreasing overall cognitive ability, perceptual reasoning skill, and auditory working memory index; however, one child with severe WS injury (score of four) showed higher average cognitive abilities. Further studies with large sample size are required to elucidate the clear correlation between the severity of WS injury and cognitive outcomes in adolescence.

Our findings suggest that the WS pattern of neonatal injury could affect the perceptual reasoning skills, and auditory working memory in late school-aged children, which may be associated with brain maturation in development. The Perceptual Reasoning Index is used to evaluate visual perception, visuospatial processing and organization, and visual–motor integration controlled by networks that include not only the posterior visual pathway but also other cortical and subcortical areas such as the frontal, temporal, and parietal lobes.^27,28^ The brain areas involved in auditory working memory are a higher-order area of the frontal cortex and auditory cortex.^29^ The delayed maturation of the frontal lobe and its ongoing connections through adolescence compared with the other brain areas are likely to be accountable for the decreased perceptual reasoning skills and auditory working memory, which could not be detected in the cohort of the present study until late school age in children with WS injury.^30^ Further functional neuroimaging studies should be conducted to elucidate the association between the pattern of injury and specific cognitive dysfunction.

In school-aged children with neonatal encephalopathy due to hypoxia–ischemia, the prevalence of epilepsy has been reported to be 10–30%.^5,26^ Two children (13%) in our study developed post-neonatal epilepsy and cerebral palsy. Both children had definite adverse cognitive outcomes in comparison to those without seizures and functional motor deficits. These findings are in keeping with prior studies that show high rates of comorbid epilepsy, cerebral palsy, and intellectual disability after hypoxic–ischemic encephalopathy.^26,31,32^ It is uncertain whether the intellectual disabilities in these children are due to the initial brain injury or whether they may be modified by epilepsy, epilepsy treatment, or motor disability.

Our study has several limitations. First, this study is a single-center study with a small sample size. Prospective studies from larger cohorts are required to provide more information about the long-term cognitive outcomes in children with neonatal encephalopathy. However, our study is meaningful considering that it is difficult to follow-up patients with a history of neonatal encephalopathy for a long period of time. Second, different neuropsychologic tests or versions were conducted to examine the cognitive functions in each participant. Our prospective cohort study has been going on over 20 years; therefore, applicable test versions have changed over time. Third, none of the patients were treated with therapeutic hypothermia since all were enrolled and treated for neonatal encephalopathy prior to implementation of therapeutic hypothermia at our center. Fourth, owing to the smaller number of participants, the neurocognitive outcomes in adolescents with BG/T injury were not evaluated in detail as compared to those with the WS injury and normal neonatal imaging. Fifth, parental education level for the cohort was high; larger studies with diverse participants are needed to better understand the role of socioeconomic status on outcomes following early brain injury. Finally, we did not have a matched control group in this study.

In conclusion, the present study suggests that adolescents with a history of neonatal encephalopathy and WS pattern of injury can have problems in their overall cognition, especially in perceptional reasoning skills and auditory working memory. Although cognitive issues may not always be evident at younger ages, we speculate that differences in overall cognitive ability in adolescence may be related to early language difficulties seen in children with WS injury due to neonatal encephalopathy. We recommend that children with neonatal encephalopathy caused by presumed hypoxic–ischemia, especially those with brain injury, be monitored carefully throughout childhood and adolescence and that there be a low threshold for psychoeducational testing through health centers or the school. Further studies should be conducted with a focus on the long-term cognitive effects of therapeutic hypothermia in children with neonatal encephalopathy; understanding the pattern and extent of brain injury may help in planning targeted interventions to improve cognitive outcomes in late childhood and adolescence.
